# Healthcare Access and Diagnostic Imaging Care Pathways for Musculoskeletal Conditions in Canada: A Cross-Sectional Analysis of the Canadian Community Health Survey 2022

**DOI:** 10.7759/cureus.107615

**Published:** 2026-04-23

**Authors:** Chidiogo J Mamah, Akinyele Oladimeji, Ezinne Ahams, Hope E Maduemezia, Nonso Ariahu, Stella Adelola, Echezona O Ukaejiofo

**Affiliations:** 1 Radiology, Afe Babalola Multisystem Hospital, Ado Ekiti, NGA; 2 Family Medicine, Obafemi Awolowo University, Ile-Ife, NGA; 3 Family Medicine, Willowgrove Medical Clinic, Saskatoon, CAN; 4 Family Medicine, eDokita Medical Clinic, Calgary, CAN; 5 Medicine and Surgery, College of Medicine, University of Lagos, Lagos, NGA; 6 Medicine, International University of Health Sciences, Basseterre, KNA; 7 General Practice, College of Medicine, University of Nigeria, Enugu, NGA

**Keywords:** canadian community health survey, chronic conditions, healthcare utilization, musculoskeletal disorders, primary care access, primary care continuity

## Abstract

Background: Musculoskeletal conditions represent a major source of disability and healthcare use worldwide. Access to primary care plays an important role in the clinical evaluation and management of these conditions, including decisions related to diagnostic assessment and referral. Understanding factors associated with access to a regular healthcare provider may help identify population groups that experience differences in primary care engagement.

Objective: This study aims to examine healthcare access and utilization among Canadians with musculoskeletal conditions and identify demographic and health-related factors associated with having a regular healthcare provider.

Methods: This cross-sectional study used data from the 2022 Canadian Community Health Survey public use microdata file. The analysis included 19,460 respondents aged 18 years and older reporting musculoskeletal conditions, representing approximately 6.8 million Canadians after application of survey weights. Descriptive statistics were calculated using weighted counts and row percentages. Survey-weighted logistic regression was used to evaluate factors associated with having a regular healthcare provider while accounting for the complex survey design.

Results: Most respondents reported having a regular healthcare provider. Higher odds were observed among those aged ≥ 65 years (adjusted odds ratio: 1.61, 95% confidence interval: 1.04-2.50, p = 0.033), those with overweight or obesity (1.19, 1.04-1.36, p = 0.012), and those with hypertension (1.29, 1.13-1.48, p < 0.001). Lower odds were associated with cardiovascular disease (0.77, 0.64-0.92, p = 0.004) and disability (0.75, 0.65-0.86, p < 0.001). Other variables were not statistically significant.

Conclusion: This study highlights differences in primary care access among Canadians with musculoskeletal conditions. Identifying groups with lower access to regular providers may help guide strategies aimed at improving continuity of care and supporting appropriate diagnostic evaluation.

## Introduction

Musculoskeletal (MSK) conditions are one of the most prevalent conditions across the world to make the burden of disease compilation, which causes pain, disability, and medical consumption [1,2]. One in five Canadians experiences chronic pain such as fibromyalgia, arthritis, and neuropathy, which costs the Canadian economy up to $60 billion a year in health care costs and lost wages and taxes, more than cardiovascular disease, cancer, and diabetes combined [3]. This is because increasing prevalence with age, coupled with the accumulation of joint damage with disease duration, has contributed to the common belief that the effects of arthritis are maximized in people who have lived long enough [4,5]. The integration of digital solutions has made it easier to diagnose, prognose, and assess rheumatic and musculoskeletal diseases [6].

In most instances, MSK disorders are first presented at family medicine clinics, and this is the initial point of care, where providers deal with a variety of MSK problems and adapt to gender-specific differences, particularly in areas with few resources [7]. The professionals working in health care have been found to have a powerful impact on the attitudes and beliefs of the patients, and it can be assumed that the conversations at a consultation can impact patient expectations [8]. One can consider a patient history and physical examination as clinician-intensive elements of the clinical encounter, as they presuppose that the clinician has to obtain the information in the examination room [9]. Clinical decision-making is, in most instances, supported by diagnostic imaging, which may include X-rays, computed tomography (CT), or magnetic resonance imaging (MRI) [10]. Imaging may aid in the detection of abnormal structures, rule out suspected diagnoses, assess disease progression, and assist with treatment planning [11]. For example, fractures, degenerative joint disease, spinal disorders, and other musculoskeletal pathologies are often diagnosed with the help of imaging [12].

Access to diagnostic imaging is usually preceded by a patient engaging with the healthcare system at the primary care level [13]. In the publicly funded healthcare system of Canada, general practitioners (GPs) or family physicians are usually the initial contact for most health concerns [14]. They become the gatekeepers of specialized services, such as referrals to diagnostic imaging and consultations with specialists, such as orthopedic surgeons or rheumatologists [15]. In turn, accessibility to a regular healthcare provider has become an important entry point in the diagnostic process of musculoskeletal conditions [16].

Even though Canada has a universal healthcare system, inequity in access to healthcare is a concern [17]. The prevalence of such a phenomenon as having a regular healthcare provider and healthcare use can depend on continuity of care, the type of provider, health status of the patients, their education, sex, age, income, and racial identity [18]. The problem of shortage of family physicians is being experienced in Canada, fueled by imposing expectations on family physicians, insufficiency of support and resources, outdated physician payment, and excessive clinic operating expenses [19]. Overutilization of diagnostic imaging is one of the primary causes of unwarranted expenditures and resource consumption in MSK condition management by the health system [20].

The objective of the study is to examine healthcare access and utilization among Canadians with MSK conditions and identify demographic and health-related factors associated with having a regular healthcare provider using data from the Canadian Community Health Survey (CCHS) 2022. The findings of the study will create a better understanding of the factors that influence this first step in the imaging and diagnostic pathway.

## Materials and methods

Study design and data source

This study used a cross-sectional design based on data from the 2022 cycle of the Canadian Community Health Survey [21]. The Canadian Community Health Survey is a nationally representative survey conducted by Statistics Canada to collect information on health status, health conditions, and health service use among individuals aged 12 years and older living in private households across Canada. The survey uses a multistage, stratified sampling design to ensure representation across provinces and territories. The 2022 dataset includes detailed information on demographic characteristics, chronic health conditions, and healthcare utilization. These data provide an opportunity to examine patterns of healthcare access among individuals reporting MSK conditions at the population level.

Study population

The analytic sample consisted of respondents aged 18 years and older who reported a MSK condition in the Canadian Community Health Survey 2022 dataset. MSK conditions were identified using the survey indicator for chronic conditions related to the bones, joints, or muscles. Respondents younger than 18 years were excluded because several health and healthcare access variables in the survey are defined for adult populations. After applying these criteria, the final analytic sample included 19,460 respondents. When survey weights were applied, this sample represented approximately 6,801,134 Canadians living with MSK conditions.

Variables and measures

The primary outcome variable was access to a regular healthcare provider. This variable was derived from the survey question asking respondents whether they had a regular medical doctor or healthcare provider. Responses were categorized as having a regular healthcare provider or not. The main explanatory variables included demographic and health-related characteristics available in the dataset. Sex was categorized as male or female. Age was categorized into four groups: 18-34 years, 35-49 years, 50-64 years, and 65 years or older. Body mass index classification was derived from the survey variable based on self-reported height and weight, and categorized as underweight or normal weight and overweight or obese according to international body mass index classification standards used in the survey. Chronic health conditions were included as binary variables indicating the presence or absence of diabetes, hypertension, high cholesterol, mood disorder, and cardiovascular disease based on self-reported diagnoses. Disability status was measured using the Washington Group disability indicator available in the survey and was categorized as no disability or at least one disability based on reported functional difficulties across domains.

Missing data

The extent of missing data was examined for all variables included in the analysis. Body mass index classification had the highest proportion of missing responses, with approximately 4.6% of observations missing. As the proportion of missing data was small and the survey design already included nonresponse adjustments in the sampling weights, a complete case approach was used for analyses involving body mass index classification. All other variables reported no missing data and were included as reported in the dataset.

Statistical analysis

All analyses accounted for the complex survey design of the Canadian Community Health Survey, including sampling weights and bootstrap replicate weights provided by Statistics Canada. The survey design variables were incorporated using the survey estimation procedures available in Stata version 18 (Stata Corp, College Station, TX) [22]. Descriptive statistics were calculated to summarize participant characteristics. Weighted counts and weighted row percentages were reported for categorical variables.

Survey-weighted logistic regression models were used to examine factors associated with having a regular healthcare provider. Adjusted odds ratios and 95% confidence intervals were estimated while controlling for sex, age group, body mass index classification, diabetes, hypertension, high cholesterol, mood disorder, cardiovascular disease, and disability status. Multicollinearity among explanatory variables was assessed using variance inflation factors. Variance inflation factor values ranged from 1.05 to 1.12 (mean: 1.07). Values close to 1 indicate minimal correlation between predictors, suggesting that multicollinearity was not a concern in the model.

Ethical considerations

This study used publicly available data from the Canadian Community Health Survey public use microdata file. The dataset contains de-identified information and does not include personal identifiers. Access to the data is governed by the Statistics Canada Open License, which permits analysis for research and educational purposes. As the data are anonymized and publicly accessible, formal research ethics board approval was not required for this analysis.

## Results

Table 1 presents the descriptive characteristics of Canadians with MSK conditions according to whether respondents reported having a regular healthcare provider.

**Table 1:** Participant characteristics by access to a regular healthcare provider among Canadians with musculoskeletal conditions (CCHS 2022; N = 19,460) Values represent weighted population estimates and row percentages derived from the Canadian Community Health Survey (CCHS) 2022. The analytic sample included 19,460 respondents, representing a weighted population of approximately 6,801,134 Canadians with musculoskeletal conditions. Estimates were calculated using survey weights and bootstrap replicate weights provided by Statistics Canada to account for the complex sampling design. All the estimates were generated using Stata version 18 (Stata Corp, College Station, TX) [22].

Variables	No Regular General practitioner (N = 1,409,800)	Has Regular General practitioner (N = 5,391,334)
Gender, n (%)		
Male	567,258 (22%)	2,034,951 (78%)
Female	842,542 (20%)	3,356,383 (80%)
Age group (years), n (%)		
18–34 years	72,093 (29%)	177,112 (71%)
35–49 years	214,776 (27%)	588,672 (73%)
50–64 years	453,756 (20%)	1,795,856 (80%)
≥65 years	669,176 (19%)	2,829,694 (81%)
Body mass index classification, n (%)		
Underweight/Normal weight	507,584 (22%)	1,755,850 (78%)
Overweight/Obese	848,323 (20%)	3,410,978 (80%)
Diabetes, n (%)		
No	1,223,107 (21%)	4,604,977 (79%)
Yes	186,693 (19%)	786,357 (81%)
Hypertension, n (%)		
No	940,986 (23%)	3,217,607 (77%)
Yes	468,814 (18%)	2,173,727 (82%)
High cholesterol, n (%)		
No	925,038 (22%)	3,337,880 (78%)
Yes	484,762 (19%)	2,053,454 (81%)
Mood disorder, n (%)		
No	1,162,277 (20%)	4,536,773 (80%)
Yes	247,523 (22%)	854,561 (78%)
Cardiovascular disease, n (%)		
No	1,174,171 (20%)	4,604,088 (80%)
Yes	235,629 (23%)	787,246 (77%)
Disability status, n (%)		
No disability	422,620 (19%)	1,850,314 (81%)
At least one disability	987,180 (22%)	3,541,020 (78%)

The results indicate that most individuals with MSK conditions reported having a regular healthcare provider. Among males, 567,258 (22%) reported no regular GP, and 2,034,951 (78%) reported having a regular GP. Among females, 842,542 (20%) reported no regular GP, and 3,356,383 (80%) reported having a regular GP. Differences were also observed across age groups. Individuals aged 18-34 years had the highest proportion without a regular provider, with 72,093 (29%) reporting no regular GP. In comparison, older groups showed lower proportions without a provider. Among individuals aged 65 years or older, 669,176 (19%) reported no regular GP, while 2,829,694 (81%) reported having a regular provider.

Body mass index categories showed similar patterns. Among individuals with underweight or normal weight, 507,584 (22%) reported no regular GP, and 1,755,850 (78%) reported having a regular provider. Among those classified as overweight or obese, 848,323 (20%) reported no regular GP, and 3,410,978 (80%) reported having a regular provider. For diabetes status, 1,223,107 (21%) without diabetes reported no regular GP compared with 4,604,977 (79%) who reported having a provider. Among individuals with diabetes, 186,693 (19%) reported no regular GP, and 786,357 (81%) reported having a provider.

For hypertension, 940,986 (23%) without hypertension reported no regular GP, and 3,217,607 (77%) reported having a provider. Among individuals with hypertension, 468,814 (18%) reported no regular GP, while 2,173,727 (82%) reported having a provider. Similar patterns were observed for cholesterol status. Among those without high cholesterol, 925,038 (22%) reported no regular GP, and 3,337,880 (78%) reported having a provider. Among those with high cholesterol, 484,762 (19%) reported no regular GP, and 2,053,454 (81%) reported having a provider.

Among respondents without a mood disorder, 1,162,277 (20%) reported no regular GP, and 4,536,773 (80%) reported having a provider. Among respondents with a mood disorder, 247,523 (22%) reported no regular GP, and 854,561 (78%) reported having a provider. For cardiovascular disease, 1,174,171 (20%) without cardiovascular disease reported no regular GP, while 4,604,088 (80%) reported having a provider. Among those with cardiovascular disease, 235,629 (23%) reported no regular GP, and 787,246 (77%) reported having a provider. Regarding disability status, 422,620 (19%) without disability reported no regular GP, and 1,850,314 (81%) reported having a provider. Among those reporting at least one disability, 987,180 (22%) reported no regular GP, and 3,541,020 (78%) reported having a regular provider.

Table 2 presents the results of the survey-weighted logistic regression analysis examining factors associated with having a regular healthcare provider among Canadians with MSK conditions.

**Table 2:** Factors associated with having a regular healthcare provider among Canadians with musculoskeletal conditions (CCHS 2022) Adjusted odds ratios (aOR) and 95% confidence intervals were estimated using survey-weighted logistic regression with bootstrap replicate weights to account for the complex sampling design of the Canadian Community Health Survey (CCHS) 2022. Reference categories were as follows: male (sex), 18-34 years (age group), underweight/normal weight (BMI), no diabetes, no hypertension, no high cholesterol, no mood disorder, no cardiovascular disease, and no disability. Estimates represent associations with having a regular healthcare provider among Canadians with musculoskeletal conditions. All estimates were produced using Stata version 18 (StataCorp, College Station, TX) [22]. Statistical significance is indicated as follows: *p < 0.05. CI: Confidence interval.

Variables	Adjusted Odds Ratio (95% CI)	p-value
Sex		
Female vs Male	1.12 (0.99–1.28)	0.079
Age group (years)		
35–49 years vs 18–34 years	1.03 (0.63–1.66)	0.914
50–64 years vs 18–34 years	1.48 (0.95–2.29)	0.080
≥65 years vs 18–34 years	1.61 (1.04–2.50)	0.033*
Body mass index classification		
Overweight/Obese vs Underweight/Normal weight	1.19 (1.04–1.36)	0.012*
Diabetes		
Yes vs No	1.00 (0.83–1.20)	0.990
Hypertension		
Yes vs No	1.29 (1.13–1.48)	<0.001*
High cholesterol		
Yes vs No	1.08 (0.95–1.22)	0.255
Mood disorder		
Yes vs No	0.98 (0.82–1.17)	0.817
Cardiovascular disease		
Yes vs No	0.77 (0.64–0.92)	0.004*
Disability status		
At least one disability vs no disability	0.75 (0.65–0.86)	<0.001*

The regression results show several factors associated with having a regular healthcare provider. Compared with individuals aged 18-34 years, those aged 65 years or older had higher odds of having a regular provider (aOR: 1.61, 95% CI: 1.04-2.50, p = 0.033). Individuals aged 50-64 years also showed higher odds (aOR: 1.48, 95% CI: 0.95-2.29, p = 0.080), although this association did not reach statistical significance. The association for individuals aged 35-49 years was not statistically significant.

Body mass index classification was also associated with access to a regular healthcare provider. Individuals classified as overweight or obese had higher odds of reporting a regular provider compared with those classified as underweight or normal weight (aOR: 1.19, 95% CI: 1.04-1.36, p = 0.012). Hypertension was significantly associated with healthcare access. Individuals with hypertension had higher odds of having a regular healthcare provider compared with those without hypertension (aOR: 1.29; 95% CI: 1.13-1.48, p < 0.001).

Cardiovascular disease and disability status were associated with lower odds of having a regular healthcare provider. Individuals with cardiovascular disease had lower odds of reporting a regular provider compared with those without cardiovascular disease (aOR: 0.77, 95% CI: 0.64-0.92, p = 0.004). Similarly, individuals reporting at least one disability had lower odds of having a regular healthcare provider compared with those without disability (aOR: 0.75, 95% CI: 0.65-0.86, p < 0.001). Although disability status was statistically associated with healthcare access, the absolute difference between groups was small, suggesting limited clinical significance. These findings should therefore be interpreted with caution. Sex, diabetes, cholesterol status, and mood disorder were not statistically associated with having a regular healthcare provider.

**Figure 1:**
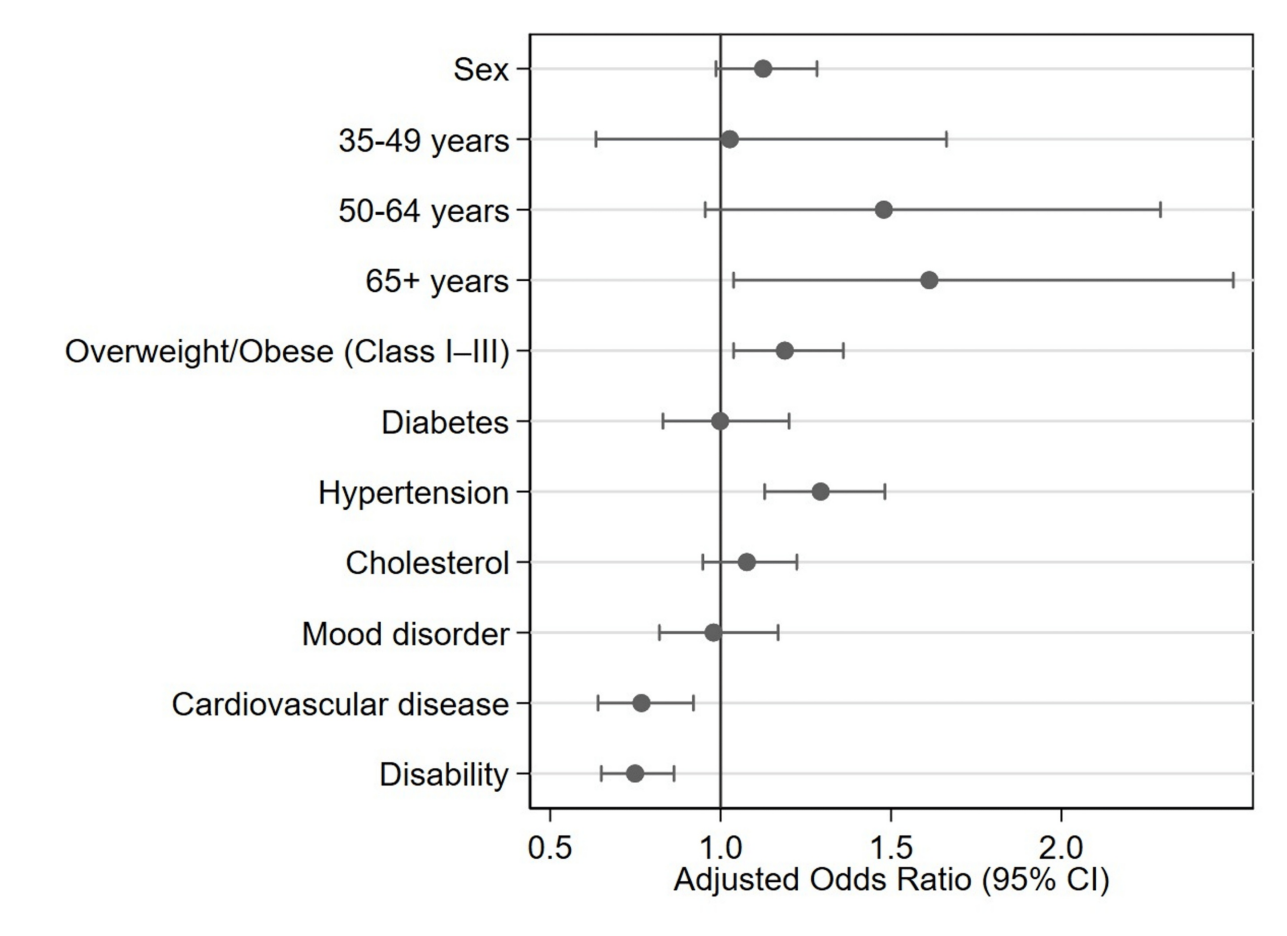
Adjusted odds ratios for factors associated with having a regular healthcare provider among Canadians with musculoskeletal conditions The vertical reference line represents an odds ratio of 1.00, indicating no association with having a regular healthcare provider.

Figure 1 illustrates the adjusted odds ratios and corresponding 95% confidence intervals for factors associated with having a regular healthcare provider among Canadians with MSK conditions.

Figure 1 visually presents the magnitude and direction of the associations reported in Table 2. Age group, body mass index classification, hypertension, cardiovascular disease, and disability status show noticeable differences in the likelihood of having a regular healthcare provider. Individuals aged 65 years or older, those classified as overweight or obese, and those with hypertension show adjusted odds ratios above 1.00. Cardiovascular disease and disability show adjusted odds ratios below 1.00. The confidence intervals for these variables do not cross the reference line, which indicates statistical significance. Other variables, including sex, diabetes, cholesterol, and mood disorder, have confidence intervals that cross the reference line, indicating that the associations were not statistically significant.

## Discussion

This study examined healthcare access among Canadians with MSK conditions and explored demographic and health-related factors associated with having a regular healthcare provider. MSK disorders encompass a range of biological mechanisms that contribute to their development, progression, and clinical impact. Central to these conditions are inflammatory processes that lead to tissue damage and pain, as well as degenerative changes within joints and MSK structures. Chronic inflammation triggers a cascade of cellular and molecular events resulting in cartilage degradation, synovial inflammation, and bone remodeling, which are hallmark features of diseases such as osteoarthritis and rheumatoid arthritis [1,2,4]. Additionally, alterations in pain pathways, including peripheral and central sensitization, contribute to persistent pain experiences beyond the initial tissue injury, reinforcing chronic pain syndromes commonly reported in MSK conditions [12]. These pathophysiologic mechanisms collectively lead to structural damage, functional impairment, and ongoing symptomatology, which underpin the high prevalence of disability observed in affected populations. The biological complexity of MSK disorders necessitates comprehensive clinical assessment and management strategies to mitigate disease progression, reduce chronic pain, and address associated disability, which in turn influences patterns of healthcare utilization and resource demands [1,2,4,12].

The results show that most individuals with MSK conditions reported having a regular provider, although access differed across several characteristics. Older adults were more likely to report having a regular healthcare provider compared with younger adults. Individuals aged 65 years or older had significantly higher odds of reporting a regular provider. Higher odds were also observed among individuals classified as overweight or obese and among those with hypertension. In contrast, individuals with cardiovascular disease and those reporting at least one disability had lower odds of having a regular healthcare provider. Although individuals with cardiovascular disease and disability may be expected to have greater healthcare engagement, the observed lower odds of having a regular healthcare provider may reflect underlying barriers to access, such as mobility limitations, system-level constraints, or unmet care needs. These findings reflect known patterns in the burden of MSK conditions and healthcare use. MSK disorders represent a major cause of pain, disability, and healthcare demand worldwide, and they often require repeated clinical assessment and management within primary care settings [1,2]. In Canada, these conditions can affect daily functioning and quality of life across different age groups, which makes access to consistent primary care important for evaluation and treatment planning [5]. Primary care providers often serve as the first point of contact for MSK complaints, where initial assessment, conservative management, and referral decisions are made [7,9]. Differences in access to a regular provider, therefore, have implications for how individuals with MSK conditions enter the healthcare system and obtain appropriate diagnostic evaluation.

Clinical practice guidelines recommend against routinely ordering imaging in patients with low back pain, and the National Committee for Quality Assurance (NCQA) Healthcare Effectiveness Data and Information Set (HEDIS) recommends that imaging for common MSK complaints, such as low back pain, should not be routinely performed during the early stages of symptoms unless there are clinical indicators of serious pathology [10]. Imaging is usually recommended only when symptoms persist or when specific warning signs are present [10]. Primary care providers play an important role in this decision process because they assess symptoms, determine whether imaging is necessary, and coordinate referrals when specialized evaluation is required. Research in primary care settings has shown that patient characteristics, provider factors, and practice environment can influence the likelihood that diagnostic imaging will be used [13]. Barriers such as uncertainty about guidelines, patient expectations, and clinical workload may also affect adherence to recommended imaging practices in primary care [20]. In this context, having access to a regular healthcare provider can support continuity of care and allow appropriate clinical assessment before imaging is considered.

Several possible mechanisms may help explain the associations observed in this study. Older adults may be more likely to have a regular healthcare provider because they often require ongoing management for multiple chronic conditions and have more frequent interactions with the healthcare system. Regular contact with primary care may increase the likelihood of establishing a continuous care relationship. Higher odds of access among individuals with hypertension may reflect similar patterns of chronic disease management, where regular follow-up and medication monitoring require ongoing contact with healthcare providers. MSK conditions frequently coexist with other chronic diseases, which can increase healthcare utilization and the need for coordinated care [4]. The association observed for body mass index classification may reflect the relationship between excess weight and MSK symptoms, which can lead individuals to seek care for joint pain, mobility issues, or functional limitations. Clinical assessment of these conditions often involves physical examination and may include diagnostic imaging when symptoms persist or when structural pathology is suspected [11,12]. In contrast, the lower odds of access observed among individuals with cardiovascular disease or disability may reflect barriers related to healthcare availability, mobility limitations, or differences in healthcare-seeking patterns. Access to primary care remains uneven in Canada, with some populations experiencing longer wait times or limited availability of family physicians [17,18]. Structural issues such as workforce shortages in family medicine may also influence the ability of individuals to establish a regular provider relationship [19]. These factors may affect how individuals with complex health needs navigate the healthcare system.

Strengths and limitations of the study

This study has several strengths and limitations that should be considered when interpreting the findings. The analysis used a large, nationally representative dataset and applied survey weights and bootstrap methods to account for the complex survey design. The dataset allowed examination of several demographic and health-related factors among Canadians with MSK conditions. At the same time, the study relied on self-reported survey data, which may introduce reporting error in health conditions and healthcare access measures. The cross-sectional design does not allow assessment of temporal relationships between variables. Some potentially relevant factors were not available in the dataset, including clinical severity of MSK conditions, physician referral patterns, and actual use of diagnostic imaging. Imaging is an important component of diagnostic evaluation for many MSK conditions, yet direct measures of imaging use were not available in the survey data. Future research using clinical datasets or administrative health records could examine how access to primary care influences diagnostic imaging decisions and care pathways for MSK conditions. Additional studies may also explore how provider availability and healthcare system factors influence access to regular care among individuals with chronic MSK disorders.

## Conclusions

This study highlights patterns of healthcare access among Canadians living with MSK conditions using nationally representative survey data. Access to a regular healthcare provider differed across age groups and health characteristics. Older adults and individuals with certain chronic conditions were more likely to report having a regular provider, while reduced access was observed among respondents with disabilities and cardiovascular disease. These findings suggest that continuity of primary care may vary across patient groups with MSK conditions. Regular access to primary care remains important for clinical assessment, monitoring of chronic conditions, and coordination of diagnostic evaluation. Future research should examine how healthcare access influences diagnostic pathways, including the use of imaging and specialist referral, using clinical or administrative datasets that capture detailed care processes.

## References

[1] Blyth FM, Briggs AM, Schneider CH, Hoy DG, March LM (2019). The global burden of musculoskeletal pain-where to from here?. Am J Public Health.

[2] Bouziri H, Roquelaure Y, Descatha A, Dab W, Jean K (2023). Temporal and spatial distribution of musculoskeletal disorders from 1990 to 2019: a systematic analysis of the global burden of disease. BMJ Public Health.

[3] Parto DN, Wong AY, Macedo L (2023). Prevalence of musculoskeletal disorders and associated risk factors in Canadian university students. BMC Musculoskelet Disord.

[4] Greggi C, Visconti VV, Albanese M (2024). Work-related musculoskeletal disorders: a systematic review and meta-analysis. J Clin Med.

[5] O'Donnell S, Rusu C, Hawker GA (2015). Arthritis has an impact on the daily lives of Canadians young and old: results from a population-based survey. BMC Musculoskelet Disord.

[6] Gupta L, Najm A, Kabir K, De Cock D (2023). Digital health in musculoskeletal care: where are we heading?. BMC Musculoskelet Disord.

[7] Dawod MS, Alswerki MN, Alelaumi AF (2025). Evaluation of musculoskeletal complaints, treatment approaches, and patient perceptions in family medicine clinics in a tertiary center in Jordan: a cross-sectional study. BMC Prim Care.

[8] Skatteboe S, Røe C, Fagerland MW, Granan LP (2017). Expectations of pain and functioning in patients with musculoskeletal disorders: a cross-sectional study. BMC Musculoskelet Disord.

[9] Keilani M, Haig AJ, Crevenna R (2016). Practical assessment in patients suffering from musculoskeletal disorders. Wien Med Wochenschr.

[10] Crowell MS, Mason JS, McGinniss JH (2022). Musculoskeletal imaging for low back pain in direct access physical therapy compared to primary care: an observational study. Int J Sports Phys Ther.

[11] Weaver JS, Omar IM, Mar WA (2022). Magnetic resonance imaging of musculoskeletal infections. Pol J Radiol.

[12] Kumar R, Marla K, Sporn K (2025). Emerging diagnostic approaches for musculoskeletal disorders: advances in imaging, biomarkers, and clinical assessment. Diagnostics (Basel).

[13] Yi SY, Narayan AK, Miles RC, Martin Rother MD, Robbins JB, Flores EJ, Ross AB (2023). Patient, provider, and practice characteristics predicting use of diagnostic imaging in primary care: cross-sectional data from the National Ambulatory Medical Care Survey. J Am Coll Radiol.

[14] Rush KL, Seaton CL, Burton L, Smith MA, Li EP (2025). The healthcare experiences of rural-living Canadians with and without a primary care provider: a qualitative analysis of open-ended cross-sectional survey responses. Prim Health Care Res Dev.

[15] Rotar AM, Van Den Berg MJ, Schäfer W, Kringos DS, Klazinga NS (2018). Shared decision making between patient and GP about referrals from primary care: does gatekeeping make a difference?. PLoS One.

[16] Goodwin R, Moffatt F, Hendrick P, Stynes S, Bishop A, Logan P (2021). Evaluation of the first contact physiotherapy (FCP) model of primary care: a qualitative insight. Physiotherapy.

[17] Lavergne MR, Bodner A, Allin S (2023). Disparities in access to primary care are growing wider in Canada. Healthc Manage Forum.

[18] Le B, Stranges S, Ali S (2025). Social and health determinants of wait times for primary care in Canada. Can Fam Physician.

[19] Li K, Frumkin A, Bi WG, Magrill J, Newton C (2023). Biopsy of Canada's family physician shortage. Fam Med Community Health.

[20] Pike A, Patey A, Lawrence R (2022). Barriers to following imaging guidelines for the treatment and management of patients with low-back pain in primary care: a qualitative assessment guided by the theoretical domains framework. BMC Prim Care.

[21] (2026). Canadian Community Health Survey: public use microdata file. https://www150.statcan.gc.ca/n1/pub/82m0013x/82m0013x2024001-eng.htm.

[22] StataCorp. 2025 (2026). Resources. https://www.stata.com/support/faqs/resources/.

